# Investigating SARS-CoV-2 Incidence and Morbidity in Ponce, Puerto Rico: Protocol and Baseline Results From a Community Cohort Study

**DOI:** 10.2196/53837

**Published:** 2024-04-19

**Authors:** Chelsea G Major, Dania M Rodríguez, Liliana Sánchez-González, Vanessa Rodríguez-Estrada, Tatiana Morales-Ortíz, Carolina Torres, Nicole M Pérez-Rodríguez, Nicole A Medina-Lópes, Neal Alexander, David Mabey, Kyle Ryff, Rafael Tosado-Acevedo, Jorge Muñoz-Jordán, Laura E Adams, Vanessa Rivera-Amill, Melissa Rolfes, Gabriela Paz-Bailey

**Affiliations:** 1 Division of Vector Borne Diseases Centers for Disease Control and Prevention San Juan Puerto Rico; 2 Faculty of Epidemiology and Population Health London School of Hygiene and Tropical Medicine London United Kingdom; 3 Ponce Research Institute Ponce Health Sciences University Ponce Puerto Rico; 4 Kāpili Services, LLC Alaka`ina Foundation Family of Companies Orlando, FL United States; 5 Faculty of Infectious and Tropical Diseases London School of Hygiene and Tropical Medicine London United Kingdom; 6 Influenza Division Centers for Disease Control and Prevention Atlanta, GA United States

**Keywords:** cohort studies, COVID-19, epidemiologic studies, Hispanic or Latino, incidence, prospective studies, research methodology, SARS-CoV-2, seroprevalence

## Abstract

**Background:**

A better understanding of SARS-CoV-2 infection risk among Hispanic and Latino populations and in low-resource settings in the United States is needed to inform control efforts and strategies to improve health equity. Puerto Rico has a high poverty rate and other population characteristics associated with increased vulnerability to COVID-19, and there are limited data to date to determine community incidence.

**Objective:**

This study describes the protocol and baseline seroprevalence of SARS-CoV-2 in a prospective community-based cohort study (COPA COVID-19 [COCOVID] study) to investigate SARS-CoV-2 infection incidence and morbidity in Ponce, Puerto Rico.

**Methods:**

In June 2020, we implemented the COCOVID study within the Communities Organized to Prevent Arboviruses project platform among residents of 15 communities in Ponce, Puerto Rico, aged 1 year or older. Weekly, participants answered questionnaires on acute symptoms and preventive behaviors and provided anterior nasal swab samples for SARS-CoV-2 polymerase chain reaction testing; additional anterior nasal swabs were collected for expedited polymerase chain reaction testing from participants that reported 1 or more COVID-19–like symptoms. At enrollment and every 6 months during follow-up, participants answered more comprehensive questionnaires and provided venous blood samples for multiantigen SARS-CoV-2 immunoglobulin G antibody testing (an indicator of seroprevalence). Weekly follow-up activities concluded in April 2022 and 6-month follow-up visits concluded in August 2022. Primary study outcome measures include SARS-CoV-2 infection incidence and seroprevalence, relative risk of SARS-CoV-2 infection by participant characteristics, SARS-CoV-2 household attack rate, and COVID-19 illness characteristics and outcomes. In this study, we describe the characteristics of COCOVID participants overall and by SARS-CoV-2 seroprevalence status at baseline.

**Results:**

We enrolled a total of 1030 participants from 388 households. Relative to the general populations of Ponce and Puerto Rico, our cohort overrepresented middle-income households, employed and middle-aged adults, and older children (*P*<.001). Almost all participants (1021/1025, 99.61%) identified as Latino/a, 17.07% (175/1025) had annual household incomes less than US $10,000, and 45.66% (463/1014) reported 1 or more chronic medical conditions. Baseline SARS-CoV-2 seroprevalence was low (16/1030, 1.55%) overall and increased significantly with later study enrollment time (*P*=.003).

**Conclusions:**

The COCOVID study will provide a valuable opportunity to better estimate the burden of SARS-CoV-2 and associated risk factors in a primarily Hispanic or Latino population, assess the limitations of surveillance, and inform mitigation measures in Puerto Rico and other similar populations.

**International Registered Report Identifier (IRRID):**

RR1-10.2196/53837

## Introduction

After the first detection of SARS-CoV-2 in late 2019, the virus has become a significant threat to health and economies globally, particularly in populations with high economic inequality and low access to health resources and infrastructure [1,2]. In the United States, racial and ethnic groups with well-documented socioeconomic disparities have also been disproportionately affected by SARS-CoV-2, with the highest COVID-19 morbidity and mortality rates among American Indian or Alaska Native, non-Hispanic Black, and Hispanic or Latino populations [3,4]. Despite tremendous progress in our understanding of SARS-CoV-2 and the development of COVID-19 treatment and vaccines, control efforts have been hindered by the emergence of new, more contagious variants and vaccine hesitancy [5-9]. A better understanding of risk and risk factors for SARS-CoV-2 infection and transmission dynamics in minority groups and low-resource settings is needed to inform ongoing control efforts and develop strategies to improve health equity.

Puerto Rico, a Caribbean island and US Commonwealth, has a high prevalence of population characteristics associated with increased vulnerability to COVID-19. Among approximately 3.3 million Puerto Rico residents, almost all (99%) identify as Hispanic or Latino, and 43% live below the federal poverty level [10]. Additionally, risk factors for severe COVID-19, including older age and chronic disease, are higher among Puerto Ricans relative to residents of the continental United States, and economic crises and natural disasters have weakened the local health care infrastructure over the past decade [11-13]. As in many regions, facility-based public health surveillance and seroprevalence data from blood banks have been and continue to be the primary sources of SARS-CoV-2 incidence and prevalence data in Puerto Rico [14-16]. While valuable, these data are limited in depth and scope due to reliance on patient health care seeking behavior and passive reporting by providers and diagnostic laboratories. Systematic diagnostic testing and follow-up of a defined population can better capture the spectrum of COVID-19 disease, determine true infection rates, and help identify risk factors for infection and morbidity.

As part of the COVID-19 emergency response efforts, the US Centers for Disease Control and Prevention (CDC) supported multiple community-based cohort studies to better understand SARS-CoV-2 transmission and risk in different settings [17-19]. The ongoing Communities Organized to Prevent Arboviruses (COPA) cohort study in Ponce, Puerto Rico—implemented in 2018 through the collaboration of the Ponce Health Sciences University, Puerto Rico Vector Control Unit, and the CDC Dengue Branch (DB)—was identified as an existing platform that could be expanded to estimate SARS-CoV-2 community infection rates and other COVID-19 research objectives in the region. In June 2020, we implemented the COPA COVID-19 (COCOVID) study, a prospective, community-based cohort to investigate the incidence of and risk factors for SARS-CoV-2 infection and morbidity among COPA participants and other residents of 15 community areas.

In this study, we describe the protocol, baseline population demographics, and seroprevalence of SARS-CoV-2 in the COCOVID cohort. The study included weekly molecular testing and multiantigen serology testing at baseline and every 6 months for SARS-CoV-2. We also collected sociodemographic data, weekly mobility and personal preventive behavior patterns, COVID-19 vaccination status, symptoms, and long-term outcomes among individuals with and without incident SARS-CoV-2 infection during the study period. To date, few longitudinal COVID-19 studies with regular SARS-CoV-2 molecular testing have included the United States or Hispanic or Latino populations [20,21], and none have been conducted in Puerto Rico. Thus, data from the COCOVID study have the potential to enhance general knowledge of SARS-CoV-2 epidemiology in addition to providing unique insights into the COVID-19 epidemic in Puerto Rico, which may have implications for prevention efforts and future epidemics in geographically or demographically similar populations.

## Methods

### Ethical Considerations

Local ethics review and approval for the COPA project and COCOVID substudy protocols were obtained through the Ponce Medical School Foundation, Inc’s institutional review board (study #171110-VR). CDC also reviewed COPA and COCOVID protocols and determined the activities were conducted in a manner consistent with applicable federal law and CDC policy (refer to 45 C.F.R. part 46; 21 C.F.R. part 56).

Adult consent, minor assent, and parental or guardian permission were obtained at enrollment and subsequent 6-month follow-up visits and documented using paper forms. The receipt of consent and assent for all participants was also confirmed daily and tracked electronically by study coordinators. All participants under the age of 21 years, the age of majority in Puerto Rico, were considered minors except for those emancipated by marriage or judicial decree. Before the interview or specimen collection, staff explained project activities and important points of informed consent to participants, answered any questions raised, and provided a paper copy of the consent form to the household. The household representative (an adult or an emancipated minor that was present at the time of visit) consented to provide household-level information and allow study staff to invite other household members to participate in the study.

All adults and emancipated minors (including household representatives) were asked to provide written consent for each of the following: providing contact information, responding to the COPA annual interview questions and providing blood and nasal swab specimens (where applicable), being contacted weekly for acute illness surveillance (AIS) and annually for cohort follow-up, responding to the COCOVID questionnaire and providing blood specimens every 6 months and nasal swabs weekly, storage of specimens remaining after testing for future studies, and permission for children for which they were the parent or legal guardian to participate in the same project activities.

The adult consent document requested a separate enumeration of each child for which they had parental or guardianship rights to account for any differences in the activities in which each child could participate. For minors aged between 14 and 20 years, written consent was obtained using a designated form simplified from the adult version. Verbal assent was documented in a designated form for minors aged between 7 and 13 years, and only parent or guardian consent was documented for minors aged between 1 and 6 years. All consent and assent forms and questionnaires were available in both Spanish and English and were written at an eighth grade or lower reading level.

This project involved the collection of personal identifying information and included safeguard measures to ensure the privacy and confidentiality of participants. Households and individual participants were assigned project-specific identifiers to track their location of residence and study status. Barcode stickers were used to link specimen tubes and paper forms to each participant’s visit during the project. All specimens were securely transported to and stored at the study office site, the CDC DB, and other laboratory sites. All personnel involved in this study were required to complete ethics training requirements set by the institutional review board and adhere to an unwavering code of conduct regarding the confidentiality of patients’ information.

Only study personnel have access to participant responses or laboratory results. Study questionnaires, laboratory test results, and other collected data are electronically stored in locations approved by the CDC for storage of personal data, including a limited-access encrypted server and shared drive within the CDC internal network, and, in the case of paper forms, in locked cabinets. Paper records will be maintained for at least 3 years and then destroyed. Where possible, deidentified data sets will be created and used for analysis, and individual participants will not be identified for any presentations or publications based on the study results.

For compensation for the time dedicated to the COCOVID study activities, participants received US $5 each week upon staff receipt of the completed weekly questionnaire and self-collected respiratory specimen, which was given in US $20 installments for every 4 successful transfers of weekly questionnaires and specimens. Participants also received US $20 at each study appointment (enrollment, 6 months, 12 months, 18 months, and 24 months) when they completed serum specimen collection and the questionnaire. If these appointments overlapped with COPA annual follow-up visits (enrollment, 12 months, and 24 months), an additional US $20 (between June 2021 and February 2022) or US $30 (between March and August 2022) in compensation was given for both activities for a total of US $40 or US $50.

### Study Overview

In June 2020, we implemented the COCOVID substudy among residents of 15 community areas in Ponce. We aimed to enroll between 900 and 1000 participants for regular surveys and specimen collection, including weekly anterior nasal swab collection for SARS-CoV-2 polymerase chain reaction (PCR) testing and venous blood draws for multiantigen immunoglobulin (Ig) G antibody testing at baseline and every 6 months. Participants were also included in activities for the larger COPA cohort for at least the same period that they were active in the COCOVID study, including weekly reporting of acute COVID-19–like symptoms through SMS text message and additional SARS-CoV-2 PCR testing among symptomatic participants.

### COPA Platform

**Figure 1:**
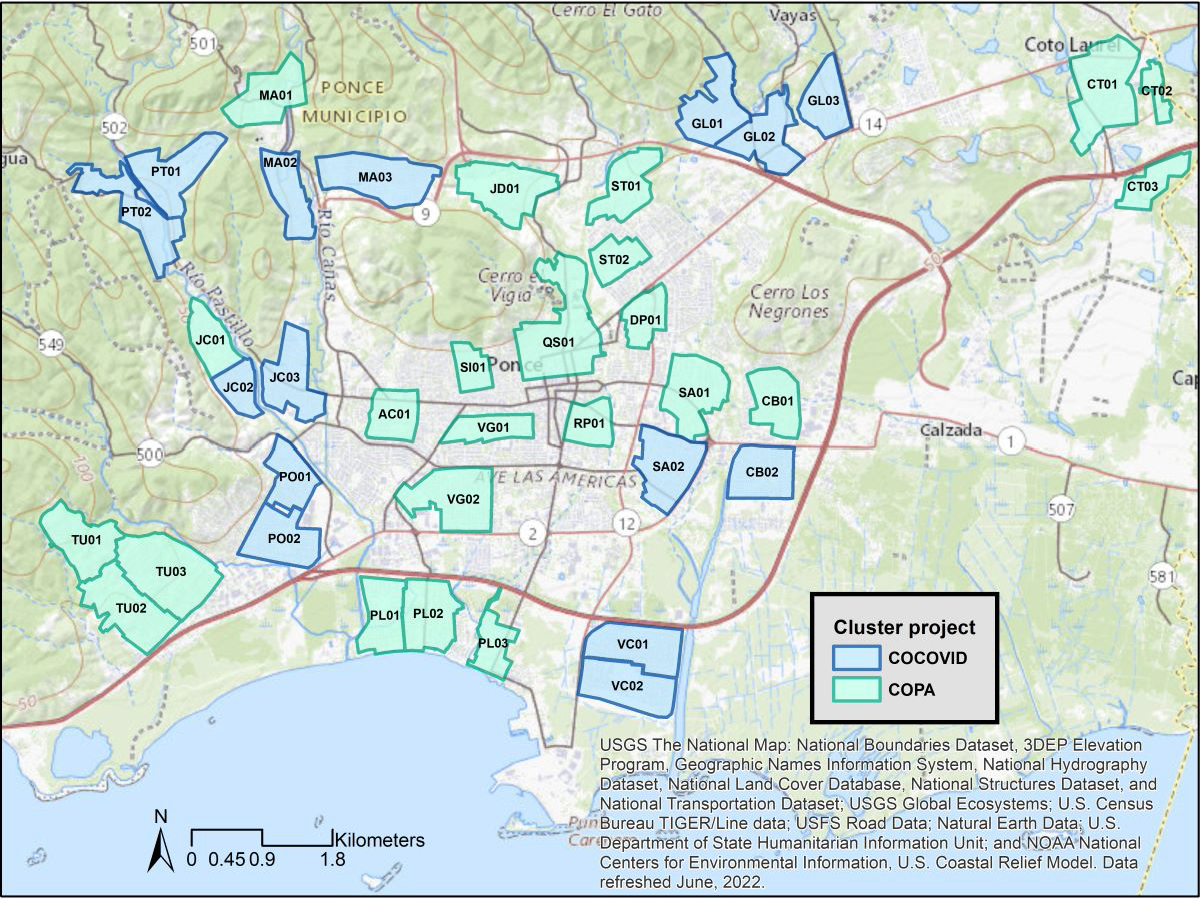
Map of the 38 Communities Organized to Prevent Arboviruses (COPA) and 15 COPA COVID-19 (COCOVID) community cohort study cluster areas in Ponce, Puerto Rico.

**Figure 2:**
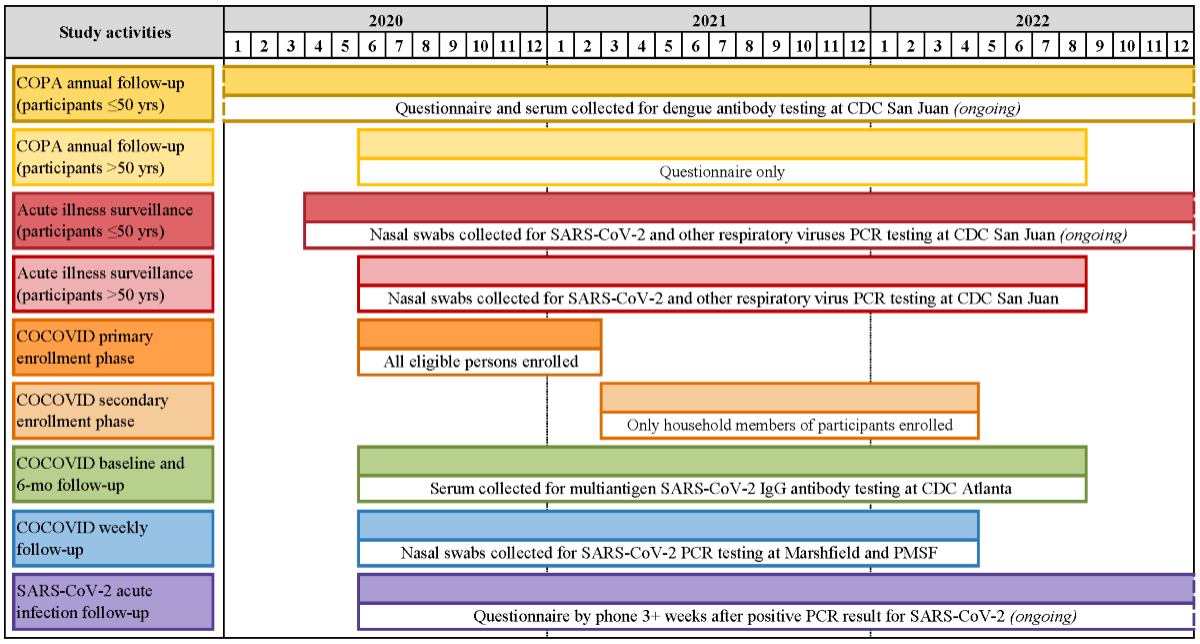
Timeline of key Communities Organized to Prevent Arboviruses (COPA) and COPA COVID-19 (COCOVID) cohort study activities in Ponce, Puerto Rico, between January 2020 and December 2022. CDC: Centers for Disease Control and Prevention; mo: months; PCR: polymerase chain reaction; PMSF: Ponce Medical School Foundation; yrs: years.

The COPA study established an ongoing cohort of approximately 3800 participants in 38 community areas in Ponce, Puerto Rico (Figure 1) to measure the incidence and prevalence of arboviral infections and evaluate vector control interventions through a cluster randomized controlled trial design [22,23]. Recruitment activities are conducted primarily through house-to-house visits, and random selections of residences in each community area are made until sample size goals are reached or all residences have been approached for recruitment. Eligible individuals are aged between 1 and 50 years, spend 4 or more nights a week in the selected residence, and have no definite plans to move in the next 12 months. Follow-up is conducted annually and includes serum collection for antibody testing for dengue and the administration of a questionnaire (Figure 2). Cohort enrollment began in March 2018.

In April 2020, questions related to risk and perceptions of COVID-19 were added to the annual questionnaire, and an AIS component was implemented (Figure 2). The AIS component is ongoing and uses an automated SMS text messaging system to contact adult participants weekly to inquire if any household members have experienced fever or other COVID-19–like symptoms (ie, cough, shortness of breath, sore throat, body pain, diarrhea, and loss of taste or smell) in the past 7 days (Figure 2). Participants that report fever are offered testing for dengue virus (DENV) by real-time reverse transcriptase–polymerase chain reaction (rRT-PCR) testing in serum, and those that report any qualifying symptom are offered an anterior nasal swab for SARS-CoV-2 and other respiratory virus rRT-PCR testing. Beginning in December 2021, participants were also asked to provide an additional anterior nasal swab for SARS-CoV-2 rapid antigen testing. To ensure the consistency of the sample collection method for both tests, all nasal swabs are collected by a nurse or medical technologist. At the time of specimen collection, project staff also administer an AIS questionnaire on recent exposures, symptoms, and health care–seeking behaviors.

### Study Population and Enrollment

Enrollment activities for the COCOVID substudy for COVID-19 began in late June of 2020. We initially selected 12 of the 38 COPA study communities for COCOVID activities, and an additional 3 community areas were added during the enrollment process to meet sample size goals (Figure 1). We prioritized the selection of community areas with high numbers of individuals aged 18 years or younger and those aged 50 years or older in COPA-participating households to increase the participation of these age groups. We also considered logistical priorities, including proximity to other selected clusters and ease of access to residential areas, in the selection process. Eligibility for COCOVID participation was expanded beyond that of COPA to include residents aged 50 years or older, in addition to spending 4 or more nights per week at a residence in a selected community area and having no definite plans to move in the next 12 months. COCOVID participants agree to participate in COPA annual follow-up questionnaires and, for participants aged between 1 and 50 years, the collection of blood specimens for arbovirus antibody testing for at least the substudy duration; participation in the AIS activities was not required for COCOVID enrollment.

Using a standardized script, study staff contacted households with 1 or more COPA participants by phone to explain the study, offer enrollment to eligible household members, and schedule an initial study visit to complete consent processes and data and specimen collection activities. Enrollment of all eligible household members was encouraged but not required, and participants were also asked to refer family and neighbors residing in the 15 community areas. The primary enrollment phase occurred between June 2020 and February 2021. Subsequently, only the enrollment of members of households with 1 or more active participants was permitted to avoid major changes to study logistics and increases in resource use (secondary enrollment phase, Figure 2). Following the completion of the primary enrollment phase, we planned to continue participants’ weekly follow-up activities for a minimum of 12 months (through February 2022) and 6-month activities for a minimum of 18 months (through August 2022).

### Sample Size Calculation

The sample size for the COCOVID substudy was based on the primary study objective of estimating community incidence of SARS-CoV-2 infection, which included allowing for comparisons by age group, accounting for potential similarity among participants within cluster areas, and assuming a cumulative incidence of infection of 10%-30% over the study period. Age groups were defined to allow infection risk comparisons of pediatric (1-17 years) and older adult (≥50 years) populations with the general population (18-49 years); gender-based sample size estimates were not made as we assumed our household-based recruitment methods would result in representative samples by gender. Our calculations assumed the enrollment of approximately equal numbers of participants across the 3 age groups and from the initially selected 12 community areas. Additionally, it was assumed that participants who were infected with SARS-CoV-2 before enrollment would not be at lower risk of further infection during the study period.

We expected a high coefficient of variation among the clusters for SARS-CoV-2 transmission, and calculations were made using a range of values from 0.5 to 0.8. We also expected a high intracluster correlation among the responses within a cluster due to the rapid spread of SARS-CoV-2, and calculations were made using values of 0.2 and 0.25. The precision of incidence is estimated by computing the 95% CI for 1 proportion in a cluster sampling design. Scenarios were run considering different cumulative incidence proportions with different intracluster correlations, coefficients of variation, and sample sizes for each cluster. Calculations showed that to reach a CI half width of 0.19 or less, a sample size of 215 participants per age group, or a total of 645 participants, was needed. To account for up to 20%-30% loss to follow-up (a high estimate to also allow for probable unequal recruitment across age groups) and an estimated 5% prevalence of previous SARS-CoV-2 infection at enrollment, we estimated that we needed to enroll a total of 900-1000 participants.

### Baseline and 6-Month Follow-Up

Baseline and subsequent 6-month visits took place at the COPA central office facilities, a designated outdoor drive-through site, or the participant’s home, depending on local social distancing regulations and participant preferences. After completing informed consent processes, study staff administered a questionnaire on chronic conditions, recent COVID-19 exposures, preventive behaviors, and, for participants that reported an acute COVID–like illness in the past 7 days, symptoms and health care seeking behaviors. Questions on COVID-19 vaccination status were added in January 2021, and questions on employment status and category, long-term COVID-19 symptoms (persisting or appearing more than 1 month after illness onset), and diagnosis of new chronic conditions after an acute SARS-CoV-2 infection were added in September 2021. A trained phlebotomist collected blood for multiantigen SARS-CoV-2 IgG antibody testing by venipuncture using aseptic techniques. Approximately 7.5 milliliters of blood were collected from participants aged between 1 and 4 years, and approximately 15 milliliters from participants aged 5 years or older.

To reduce study burden, COPA annual activities were conducted at the same time as COCOVID baseline and 6-month follow-up visits, where possible (Figure 2). In these cases, an additional ~7.5 mL of blood for dengue antibody testing were collected during the COCOVID visit for participants aged between 1 and 50 years, and the COPA annual questionnaire was administered to all participants by phone. This questionnaire included questions about household composition and income, demographics, employment status, and COVID-19 risk perception. The 6-month follow-up activities were conducted through August 2022 (Figure 2).

### Weekly Follow-Up

Each week, participants self-collected (or had a parent or guardian assist them in collecting) a flocked swab of the anterior nares (anterior nasal swab) and completed a paper questionnaire on new or worsening symptoms, illness among household members, and behaviors such as leaving their home for activities with people outside of their household, places visited, mask use, and social distancing. Before distribution, a unique barcode label was applied to a specimen collection tube and corresponding weekly questionnaire, and questionnaires were prefilled with the participant’s name, date of birth, and study identifier. At the enrollment appointment, the first weekly nasal swab specimen and accompanying questionnaire were completed under study staff instruction and supervision, and an initial 4-week supply of paper questionnaires and nasal swab specimen collection supplies were provided. Participants were asked to collect nasal swab specimens and fill out the accompanying questionnaire on a consistent day of the week within 0-12 hours of a scheduled transfer to study staff.

Study staff drove to the participating houses on an agreed-upon day and time for specimen and questionnaire pick-up each week. Households that were unavailable for scheduled pick-up (incidentally or always) were asked to drop off collected specimens and questionnaires at an agreed-upon day and time at the COPA office site. An automated SMS text-messaging system through Twilio MessagingX (Twilio, Inc) was used to send reminders to households on the day of their scheduled pick-up or drop-off for respiratory specimen collection, and study staff contacted participants by phone within 0-2 days if specimen and questionnaire receipt was delayed without notice and to resolve other issues related to received specimens and questionnaires, such as specimen leaks and missing responses. If scheduled receipt of a weekly respiratory specimen was delayed by 4 or more days, no additional opportunities for specimen transfer were offered, and the specimen was considered missing for that week. Additional 4-week supplies of respiratory specimen collection kits and questionnaires were distributed to participants as needed during pick-up and drop-off activities.

Weekly follow-up activities were initially planned to conclude in February 2022, after 12 months of the end of the primary enrollment period. However, investigators agreed to extend the weekly follow-up period due to an increase in SARS-CoV-2 transmission following the introduction of the Omicron variant in Puerto Rico. Study materials and funds were identified to permit a 2-month extension of weekly follow-up to a total of 98 weeks from June 2020 to April 2022 (Figure 2).

### Follow-Up for Participants With Symptoms or SARS-CoV-2 Infection

Participants that reported fever, cough, shortness of breath, sore throat, body pain, diarrhea, or loss of taste or smell in the weekly questionnaires (or through the AIS SMS text message system) were asked to participate in a COPA AIS visit to collect additional samples for expedited testing for dengue, SARS-CoV‑2, and other respiratory viruses and answer the AIS questionnaire.

Participants with a positive SARS-CoV-2 molecular test from nasal swab specimens collected during weekly COCOVID follow-up or as part of AIS were contacted by phone at least 4 weeks after specimen collection (depending on delays in receipt of positive test results) to complete a follow-up questionnaire on symptoms and illness duration, health care seeking behavior, travel, and other exposures before infection, and to take measures to prevent household transmission while sick (Figure 2).

### Specimen Storage, Transport, and Testing

Participants were asked to store weekly anterior nasal swab specimens in their home refrigerators until they were transferred to study staff. Upon receipt of nasal swab specimens, staff stored them in the COPA laboratory refrigerator at 4 °C or according to current CDC specimen storage guidelines. Staff centrifuged whole blood specimens within 4 hours of collection to separate out serum and stored them in the COPA laboratory refrigerator until transport. Specimens were transported by a courier 3 times weekly from the COPA office site to the CDC DB laboratory in San Juan, Puerto Rico, for accessioning, processing, and storage at –70 °C. Frozen nasal swab specimens from COCOVID weekly collection activities were shipped every 1-3 months, and serum aliquots from COCOVID 6-month collection were shipped every 3-6 months to designated laboratories for testing.

Weekly nasal swab specimens were tested by multiplex rRT-PCR assays to detect SARS-CoV-2 RNA, including the Quidel Lyra SARS-CoV-2 Assay [24], Thermo Fisher Scientific TaqPath COVID-19 Combo Kit [25], Thermo Fisher Scientific TaqPath COVID-19, FluA, FluB Combo Kit [26], and Roche Cobas SARS-CoV-2 assay [27]. Cycle threshold values for assay targets were provided for positive specimens, and overall test result interpretations were provided for all tested specimens. Specimens collected between June 2020 and February 2022 were tested by Marshfield Labs in Marshfield, Wisconsin; those collected between March and April 2022 were tested by the Ponce Medical Sciences Foundation Immunology Reference Laboratory in Ponce, Puerto Rico. Whole genome sequencing is ongoing and being performed at CDC headquarters (Atlanta, Georgia) or DB laboratories for weekly nasal swabs that tested positive by rRT-PCR and have sufficiently low cycle threshold values for the nucleocapsid (N) protein target (≤30 for headquarters and ≤27 for DB laboratories). Information on the detected variant lineage (through Pangolin COVID-19 Lineage Assigner software version 3.1.7; Centre for Genomic Pathogen Surveillance) is provided in accordance with CDC guidance [28].

Serum specimens collected for baseline and 6-month follow-up visits were tested by the Luminex xMAP SARS-CoV-2 multiantigen IgG assay to detect previous infection with SARS-CoV-2 [29]. This assay has 3 antigen targets: the N structural protein, the S1 subunit, and the receptor binding domain (RBD), which are part of the spike (S) protein. Interim guidelines for SARS-CoV-2 testing from the US CDC recommend using anti-N IgG antibody testing to evaluate previous SARS-CoV-2 infection in vaccinated individuals, as COVID-19 vaccines are designed to encode the S protein or a portion of it. Thus, to reduce potential misclassification due to vaccination versus past SARS-CoV-2 infection, specimens were only determined to be positive if IgG antibodies against the N protein, in addition to those against at least 1 of the other 2 targets (S1 and RBD), were detected. The CDC Microbial Pathogenesis and Immune Response Laboratory in Atlanta, Georgia, conducted testing on all serum specimens collected during the study period.

Serum and nasal swab specimens collected for AIS activities were tested at the CDC DB laboratory. Serum samples were tested by the CDC DENV-1-4 rRT-PCR Multiplex Assay to detect RNA for DENVs 1, 2, 3, and 4 and by the DENV Detect IgM Capture enzyme-linked immunosorbent assay kit to detect IgM antibodies for DENV antigens [30,31]. Anterior nasal swabs were tested by an in-house respiratory virus rRT-PCR multiplex assay to detect RNA for SARS-CoV-2, influenza A and B viruses, parainfluenza viruses, adenovirus, respiratory syncytial virus, and human metapneumovirus. The DENV and respiratory virus rRT-PCR assays for AIS specimens were run weekly or more often, and participants with positive results were notified by phone and given recommendations on preventing transmission and following up with their health care providers as needed.

### Data Collection and Management

#### Baseline and 6-Month Questionnaire

Between June 2020 and February 2022, the EpiInfo mobile app was used on Samsung tablets to administer the baseline and 6-month questionnaires and record responses, including participant identifiers and specimen collection data [32]. Beginning in March 2022, the Research Electronic Data Capture (REDCap; Vanderbilt University) mobile application was used to administer these questionnaires [33,34]. Paper questionnaire forms were available for use as a backup in case of electronic data collection failure. Paper forms were entered into the mobile app either daily or every 6 months as part of the data review and cleaning processes. Questionnaire data were transferred daily from tablets to a secure, limited-access CDC server through the Secure Shell File Transfer Protocol.

#### Weekly Questionnaire

A paper version of the weekly questionnaire was completed by participants and received by study staff at scheduled specimen pick-up and drop-off activities. Staff reviewed the form for completeness and filled out the laboratory section to accompany the corresponding weekly nasal swab specimen. After the specimens were transported to and accessioned by the CDC DB laboratory, the forms were scanned into the CDC laboratory samples database, where they underwent automated data extraction using Microsoft Azure Form Recognizer software or manual data entry in cases of software extraction failure [35].

Preliminary data cleaning was performed in EpiInfo or REDCap daily by study field staff and the data quality team. Additional data cleaning processes were performed weekly using the R environment for statistical computing (R Core Team) and involved data validation and standardization, including the identification of missing forms, data inconsistencies, and variable completeness [36]. Final data checks and standardizations were also performed using R statistical software at 6-month intervals to generate analytic data sets.

### Data Analysis

#### Outcome Measures

The primary outcome measures of interest for this study are (1) the incidence of SARS-CoV-2 infection as defined by detection of SARS-CoV-2 RNA by PCR testing of participant specimens collected as part of weekly follow-up or AIS visits, and (2) the relative risks of incident SARS-CoV-2 infection by participant demographics, health history, and behaviors. Secondary outcomes included the (1) seroprevalence of SARS-CoV-2 defined by detection of N and either S1 or RBD IgG antibodies by enzyme-linked immunosorbent assay, (2) household attack rate among households with at least 2 participants and 1 participant with an incident SARS-CoV-2 infection, and (3) the frequency of illness characteristics and outcomes (ie, symptom status and severity, health care seeking, death, and post–COVID conditions) among participants with incident SARS-CoV-2 infections.

#### Planned Statistical Analyses

##### SARS-CoV-2 Infection Incidence and Risk Factors

Using the number of incident SARS-CoV-2 infection events detected through PCR testing divided by the total person-time at risk contributed, we calculated SARS-CoV-2 infection incidence rates overall and approximately every 6 months during the study period (between June 2020 and August 2022). We will also estimate the incidence rates separately for primary and secondary infections, during the circulation of major SARS-CoV-2 variants, and by COVID-19 vaccination status. We will use Poisson regression to estimate adjusted infection risks by age group, primary SARS-CoV-2 variant circulating at the time of infection, previous SARS-CoV-2 infection status, and COVID-19 vaccination status, and identify other potential risk factors for infection in our cohort. Household will be accounted for as a clustering effect in the regression, using the sandwich estimator framework to calculate a clustered SE [37]. We will extrapolate the cumulative incidence of SARS-CoV-2 infections in the cohort to the general population in the Ponce region during the study, using population age and sex estimates from US census data numbers, and compare this extrapolated estimate to surveillance data from the same time period.

##### SARS-CoV-2 Infection Seroprevalence

Using the overall interpretation for the SARS-CoV-2 IgG antibody testing (positive only when N and either S1 or RBD antibodies are detected), we will estimate the seroprevalence of SARS-CoV-2 infection among participants of the COCOVID cohort approximately every 6 months during the study period, using specimens collected during those periods. The frequency of seroconversion, defined as a positive SARS-CoV-2 IgG antibody test result following a negative result, will be assessed over the study period at the individual and household level. For the period during which both PCR and antibody testing were occurring (between June 2020 and April 2022), we will compare the proportion of participants with seroconversion detected with SARS-CoV-2 incidence estimates from weekly PCR testing. This comparison will be used to evaluate the sensitivity and specificity of repeated serology testing at 6-month intervals in estimating infection incidence. We will also use the results of this comparison to inform estimates of SARS-CoV-2 infection incidence in the cohort that occurred between May and August 2022, when only SARS-CoV-2 IgG test results were available.

##### Household Attack Rate

We will assess potential household transmission of SARS-CoV-2 among households with at least 2 participating members and 1 participating member with an incident SARS-CoV-2 infection (index case) during study follow-up. Additional household cases will be defined based on the detection of SARS-CoV-2 RNA by PCR testing in a household member within 14 days of the index case. We will calculate a crude household attack rate from the proportion of additional cases among all household members deemed susceptible to acquiring SARS-CoV-2 infection from an index case. To account for household transmissions from nonindex cases and identify factors associated with household transmission, we will consider using a chain binomial transmission model to estimate the household attack rate and identify factors associated with increased and decreased likelihood of intrahousehold transmission [38,39].

##### Frequency of Illness Characteristics and Outcomes

Among COCOVID participants with incident infections, we will assess the frequency of symptomatic versus asymptomatic infection, self-reported symptoms, illness duration, health care seeking behaviors, and outcomes, including uncomplicated recovery, hospitalization, death, and the development of post–COVID conditions. If sample size permits, we propose using robust Poisson regression models to identify participant demographics, symptoms, and comorbidities associated with seeking care and being diagnosed with COVID-19, severe disease, and the development of self-reported post–COVID-19 conditions [40]. We will compare acute illness events reported in COCOVID weekly questionnaires associated with an incident SARS-CoV-2 infection to those not associated with SARS-CoV-2 infection to identify symptoms and symptom combinations that are highly indicative of infection [41].

#### Statistical Analyses of Baseline Data

In this study, we describe the baseline characteristics of the COCOVID cohort using data collected from questionnaires applied at COCOVID enrollment or within the previous year for participants previously enrolled in COPA. Frequency statistics are presented overall for the COCOVID cohort and compared to the populations of Ponce and Puerto Rico based on data from the US Census American Community Survey 2021 1-Year estimates [10,42]. Pearson chi-square tests were used for comparisons of the cohort population with those of Ponce and Puerto Rico at a household and individual level; multiple comparisons were conducted for variables with more than 2 categories, and Bonferroni-adjusted *P* values are presented. For the cohort population, individual frequency statistics were also presented by serological evidence of previous SARS-CoV-2 infection at baseline, as indicated by IgG antibody testing by timing of study enrollment, age group, sex, and annual household income. Pearson chi-square or Fisher exact tests, in the case of 1 or more expected values of <5, were used to compare categorical variables by baseline serostatus. A significance level of .05 was assumed for all comparisons, including following Bonferroni adjustment where applicable.

All analyses were conducted using R (version 4.0.4; R Core Team) [36].

## Results

During the first phase of enrollment (between June 2020 and February 2021), a total of 1012 individuals from 388 households were enrolled in the COCOVID cohort; an additional 18 members of participating households were enrolled in the secondary enrollment phase, with the last participant enrolled in November 2021. Among 388 households with at least 1 COCOVID participant, 83.03% (1042/1255) of all household members were enrolled, with a mean of 3.2 participants per household; 40.27% (151/375) of households had an annual income under US $20,000 (Table 1). Of the 1030 total participants, 56.21% (579/1030) had previously participated in the COPA study, the mean age was 35.9 (range 1-97) years, and 53.40% (550/1030) were female (Table 2). Most participants identified as Latino/a (1021/1025, 99.61%) and Puerto Rican (1015/1024, 99.12%), and around two-thirds (682/1016, 67.13%) identified as White. There were 719 adult participants (aged 21 years or older; 69.8%) enrolled, of which approximately two-thirds had college or technical school degrees (197/716, 27.51%) or postgraduate study (278/716, 27.36%) and half were employed (380/717, 53.00%). More than half (389/719, 54.10%) of adult participants and 45.66% (463/1014) of all participants reported having been diagnosed with at least 1 chronic health condition.

**Table 1:** Annual income of households with ≥1 participant in the COPA COVID-19 (COCOVID) cohort study at baseline, between June 2020 and November 2021, compared to those of the Ponce municipality and Puerto Rico populations according to 2020 and 2021 US Census American Community Survey estimates.

Annual household income (US $)^a^	Households with ≥1 COCOVID participant (N=388), n (%)	Households in Ponce municipality (N=50,007), n (%)	*P* value^b^	Households in Puerto Rico (N=1,165,982), n (%)	*P* value^b^
<10,000	61 (16.3)	16,659 (33.3)	<.001	286,499 (24.6)	<.001
10,000-19,999	90 (24)	9,783 (19.6)	.16	250,325 (21.5)	>.99
20,000-29,999	86 (22.9)	6,792 (13.6)	<.001	176,614 (15.1)	<.001
30,000-49,999	90 (24)	7,719 (15.4)	<.001	208,722 (17.9)	.01
>50,000	48 (12.8)	9,054 (18.1)	.04	243,822 (20.9)	<.001

^a^Participants or households who declined to respond or were missing a response were excluded from the denominator in percentage calculations (<4%).

^b^*P* values for characteristics with more than 2 categories were adjusted for multiple comparisons by Bonferroni correction.

**Table 2:** Demographics of all participants in the COPA COVID-19 (COCOVID) cohort study at baseline, between June 2020 and November 2021, compared to those of the Ponce municipality and Puerto Rico populations according to 2020 and 2021 US Census American Community Survey estimates.

		COCOVID study participant (N=1,030), n (%)	Ponce municipality residents (N=135,084), n (%)	*P* value^a^	Puerto Rico residents (N=3,263,584), n (%)	*P* value^a^
**Age group (years)**						
	1-9	75 (7.3)	11,105 (8.2)	>.99	254,038 (7.8)	>.99
	10-17	174 (16.9)	12,603 (9.3)	<.001	291,750 (8.9)	<.001
	18-24	119 (11.6)	12,823 (9.5)	.20	316,387 (9.7)	.35
	25-34	129 (12.5)	17,660 (13.1)	>.99	402,204 (12.3)	>.99
	35-44	181 (17.6)	14,827 (11)	<.001	400,607 (12.3)	<.001
	45-54	198 (19.2)	15,391 (11.4)	<.001	415,974 (12.7)	<.001
	55-64	52 (5.1)	17,600 (13)	<.001	442,138 (13.5)	<.001
	≥65	102 (9.9)	33,075 (24.5)	<.001	740,486 (22.7)	<.001
**Female**		550 (53.4)	70,555 (52.2)	.47	1,719,593 (52.7)	.67
**Identify as Latino or Latina^b^**		1021 (99.6)	134,560 (99.6)	.80	3,239,060 (99.2)	.27
**Race^b^**						
	White	682 (68.9)	45,980 (34)	<.001	914,840 (28)	<.001
	Black	102 (10.3)	5,171 (3.8)	<.001	200,391 (6.1)	<.001
	Other	47 (4.7)	13,477 (10)	<.001	981,183 (30.1)	<.001
	Multiple races	159 (16.1)	70,456 (52.2)	<.001	1,167,170 (35.8)	<.001

^a^*P* values for characteristics with more than 2 categories were adjusted for multiple comparisons by Bonferroni correction.

^b^Participants or households who declined to respond or were missing a response were excluded from the denominator in percentage calculations (<4%).

Compared to the overall populations of both Ponce and Puerto Rico, the COCOVID cohort population differed significantly by annual household income, age group, race, education level, and employment status (Tables 1-3). Households with at least 1 COCOVID participant more frequently belonged to middle income groups of US $20,000-US $29,999 (86/375, 22.93% in cohort vs 6792/50,007, 13.58% in Ponce and 176,614/1,165,982, 15.15% in Puerto Rico) and US $30,000-US $49,000 (90/375, 24.00% vs 7719/50,007, 15.44% and 208,722/1,165,982, 17.90%), and less frequently the highest, >US $50,000 (48/375, 12.80% vs 9054/50,007, 18.11% and 243,822/1,165,982, 20.91%), and lowest, <US $10,000 (61/375, 16.27% vs 16,659/50,007, 33.31% and 286,499/1,165,982, 24.57%), income groups (*P*<.05; Table 1). The cohort had relatively high proportions of participants in the age groups of 10-17 years (174/1030, 16.89% in cohort vs 12,603/135,084, 9.33% in Ponce and 291,750/3,263,584, 8.94% in Puerto Rico), 35-44 years (181/1030, 17.57% vs 14,827/135,084, 10.98% and 400,607/3,263,584, 12.28%), and 45-54 years (198/1030, 19.22% vs 15,391/135,084, 11.39% and 415,974/3,263,584, 12.75%), and low proportions of participants in the oldest age groups, 55-64 years (52/1030, 5.05% vs 17,600/135,084, 13.03% and 442,138/3,263,584, 13.55%), and ≥65 years (102/1030, 9.90% vs 33,075/135,084, 24.48% and 740,486/3,263,584, 22.69%; *P*<.001; Table 2). Cohort participants were more frequently identified as White (683/990, 68.99% in the cohort vs 45,980/135,084, 34.04% in Ponce and 913,840/3,263,584, 28.00% in Puerto Rico) or Black (102/990, 10.30% vs 5171/135,084, 3.83% and 200,391/3,263,584, 6.14%) and less frequently as another race (47/990, 4.75% vs 13,477/135,084, 9.98% and 981,183/3,263,584, 30.06%) or mixed race (159/990, 16.06% vs 70,456/135,084, 52.16% and 1,167,170/3,263,584, 35.76%; *P*<.001). For populations 25 years and older, cohort participants were less likely to have a less than high school education (38/659, 5.77% in cohort vs 19,235/98,553, 19.52% in Ponce and 488,780/2,401,409, 20.35% in Puerto Rico) and more likely to have some college or associate degree (186/659, 28.22% vs 21,209/98,553, 21.52% and 556,795/2,401,409, 23.19%) and a bachelor’s degree or higher (268/659, 40.67% vs 31,402/98,553, 31.86% and 683,894/2,401,409, 28.48%; *P*<.001; Table 3). Additionally, cohort participants aged 25 years or older were more likely to be in the labor force relative to Ponce and Puerto Rico residents in the same age group, including in employed (356/644, 55.28% vs 35,976/98,553, 36.50% and 977,860/2,401,409, 40.72%) and unemployed groups (74/644, 11.49% vs 6037/98,553, 6.13% and 119,812/2,401,409, 4.99%; *P*<.001).

At enrollment, 1.55% (16/1030) of COCOVID participants were seropositive for SARS-CoV-2, of which the median age was 46 (IQR 33-51) years, 56.25% (9/16) were male, and half (50.00%, 8/16) reported chronic health conditions (Table 4). There was a significant difference in the timing of study enrollment among participants with and without baseline seropositivity for SARS-CoV-2, with a higher proportion of seropositive participants recruited in the later months of the study (*P*=.003). No significant differences by age group, sex, or household income were observed among participants that were seropositive and those that were seronegative at baseline.

**Table 3:** Education level and employment status of adult participants aged ≥25 years in the COPA COVID-19 (COCOVID) cohort study at baseline, between June 2020 and November 2021, compared to those of the Ponce municipality and Puerto Rico populations according to 2020 and 2021 US Census American Community Survey estimates.

		COCOVID study participants aged ≥25 years (N=662), n (%)	Ponce municipality residents aged ≥25 years (N=98,553), n (%)	*P* value^a^	Puerto Rico residents aged ≥25 years (N=2,401,409), n (%)	*P* value^a^
**Educational attainment^b^**						
	Less than high school graduate	38 (5.7)	19,235 (19.5)	<.001	488,780 (20.4)	<.001
	High school graduate or equivalent	167 (25.3)	26,707 (27.1)	>.99	671,940 (28)	.13
	Some college or associate degree	186 (28.2)	21,209 (21.5)	<.001	556,795 (23.2)	.01
	Bachelor’s degree or higher	268 (40.7)	31,402 (31.9)	<.001	683,894 (28.5)	<.001
**Employment status^b^**						
	Employed	356 (55.3)	35,976 (36.5)	<.001	977,860 (40.7)	<.001
	Unemployed	74 (11.5)	6,037 (6.1)	<.001	119,812 (5)	<.001

^a^*P* values for characteristics with more than 2 categories were adjusted for multiple comparisons by Bonferroni correction.

^b^Participants or households who declined to respond or were missing a response were excluded from the denominator in percentage calculations (<4%).

**Table 4:** Timing of study enrollment and demographics and of participants by serological evidence of previous SARS-CoV-2 infection at baseline, COPA COVID-19 (COCOVID) study, Ponce, Puerto Rico, between June 2020 and November 2021 (N=1030).

		Participants seropositive for SARS-CoV-2 at baseline (N=16), n (%)	Participants seronegative for SARS-CoV-2 at baseline (N=1014), n (%)	Prevalence of seropositivity for SARS-CoV-2 at baseline	*P* value	
**Timing of study enrollment**						.003
	June-August 2020	0 (0)	324 (32)	0		
	September-November 2020	5 (31.3)	405 (39.9)	1.2		
	December 2020–February 2021	10 (62.5)	268 (26.4)	3.6		
	March 2021 or later	1 (6.3)	17 (1.7)	5.6		
**Age group (years)**						.24
	1-17	1 (6.3)	248 (24.5)	0.4		
	18-34	4 (25)	244 (24.1)	1.6		
	35-49	5 (31.3)	301 (29.7)	1.6		
	50-64	4 (25)	121 (11.9)	3.2		
	≥65	2 (12.5)	100 (9.9)	1.9		
**Sex**						.46
	Female	7 (43.8)	543 (53.6)	1.3		
	Male	9 (56.3)	471 (46.5)	1.9		
**Annual income for household (US $)^a^**						.12
	<10,000	3 (18.8)	172 (17)	1.7		
	10,000-19,999	5 (31.3)	208 (20.6)	2.3		
	20,000-29,999	0 (0)	234 (23.2)	0		
	30,000-49,999	5 (31.3)	241 (23.9)	2		
	>50,000	3 (18.8)	133 (13.2)	2.2		

^a^Participants that declined to respond were excluded from the denominator in percentage calculations (<3%).

## Discussion

Few studies with prospective assessments of incident SARS-CoV-2 infections at the community level have been described to date, and none have been implemented in Puerto Rico or other primarily Latino or Hispanic US populations [17-19,43-45]. The COCOVID community-based cohort study provides a unique opportunity to assess SARS-CoV-2 infection incidence, risk factors, and outcomes in Puerto Rico and also help further characterize asymptomatic infections, household transmission, and serological and molecular testing dynamics. We were able to build the COCOVID study into an existing cohort study platform, allowing for rapid enrollment and the implementation of extensive data collection activities. Prospective follow-up through weekly PCR testing of participants and regular serology testing enabled comprehensive detection of SARS-CoV-2 infections within the cohort population. Additionally, our large sample size and extended follow-up period strengthened our assessment of SARS-CoV-2 incidence and outcomes by time-dependent factors, including lockdown measures, circulating SARS-CoV-2 variants, and COVID-19 vaccination status.

COCOVID surpassed its enrollment goal with 1030 participants with minimum sample size goals (n=215) reached in the 3 age groups of interest (1-17 years, 18-49 years, and ≥50 years). The cohort included participants with characteristics associated with increased COVID-19 morbidity risk [3,46,47], although sometimes with lower frequency than the general populations for Ponce and Puerto Rico. Consistent with these populations, almost all COCOVID participants identified as Latino/a. Chronic health condition prevalence was high among adults in the cohort and in accordance with estimates from the Behavioral Risk Factor Surveillance System [48]. The cohort had relatively low proportions of participants in the oldest (≥65 years) age group and households in the lowest (<US $10,000) annual income group compared to Ponce and Puerto Rico populations. However, these groups still accounted for at least 10% of cohort participants and households, respectively, which should facilitate adjusted analyses and extrapolation of results to the general population. SARS-CoV-2 seroprevalence at enrollment was low and increased among cohort participants enrolled later in the study period. The low but increasing seroprevalence of SARS-CoV-2 observed among study participants at baseline over time is consistent with surveillance data from the region [14]. As a result, there was a limited sample size to assess differences in SARS-CoV-2 seroprevalence by age group and other characteristics, and factors associated with the seroprevalence of SARS-CoV-2 infection will be explored more fully using follow-up data from the study.

We did not use a formal sampling frame, but rather our sampling strategy was determined by logistic constraints to include a limited selection of community areas with primarily single-resident homes and no public housing or large apartment buildings. Additionally, most of our participants were previously enrolled in the larger COPA cohort, whose eligibility was restricted to participants younger than 50 years. This likely led to the observed oversampling of larger, middle-income households and employed, middle-aged adults and their children relative to the overall populations of Ponce and Puerto Rico. Additionally, individuals who agreed to enroll in COCOVID (and COPA) may be more concerned about disease risks and more likely to take measures to prevent SARS-CoV-2 infection than the general populations of Ponce and Puerto Rico. However, there was diversity across sociodemographic indicators in our study population, and the detailed data collected on household and individual characteristics and preventive behaviors can be used to adjust for these potential impacts on SARS-CoV-2–related outcomes and extrapolate our findings to the general population, where possible.

Despite tremendous progress in our understanding of SARS-CoV-2 transmission and control, including the development of effective vaccines, the persistence of health inequities and other challenges underpin the need for more comprehensive data to inform mitigation measures and prevent future outbreaks. The COCOVID study provides a valuable opportunity to investigate community and household SARS-CoV-2 infections in a primarily Hispanic or Latino population with diversity across several risk factors for COVID-19. Study methodology supports thorough detection of asymptomatic and symptomatic infections to help us better estimate the burden of SARS-CoV-2 in the region and assess the limitations of surveillance, which currently remains the primary resource for SARS-CoV-2 data for the island.

## Abbreviations

AIS: acute illness surveillance
CDC: Centers for Disease Control and Prevention
COCOVID: Communities Organized to Prevent Arboviruses COVID-19
COPA: Communities Organized to Prevent Arboviruses
DB: Dengue Branch
DENV: dengue virus
Ig: immunoglobulin
N: nucleocapsid
PCR: polymerase chain reaction
RBD: receptor binding domain
REDCap: Research Electronic Data Capture
rRT-PCR: real-time reverse transcriptase–polymerase chain reaction
S: spike

## Data Availability

The data sets generated and analyzed during this study are not publicly available due to data security and confidentiality guidelines determined by the study governance board, but are available from the corresponding author upon reasonable request.
